# *Listeria monocytogenes*: The Impact of Cell Death on Infection and Immunity

**DOI:** 10.3390/pathogens7010008

**Published:** 2018-01-11

**Authors:** Courtney E. McDougal, John-Demian Sauer

**Affiliations:** Department of Medical Microbiology and Immunology, School of Medicine and Public Health, University of Wisconsin-Madison, Madison, WI 53706, USA; cmcdougal@wisc.edu

**Keywords:** *Listeria monocytogenes*, cell death, necrosis, apoptosis, pyroptosis, cell-mediated immunity, CD8+ T-cells

## Abstract

*Listeria monocytogenes* has evolved exquisite mechanisms for invading host cells and spreading from cell-to-cell to ensure maintenance of its intracellular lifecycle. As such, it is not surprising that loss of the intracellular replication niche through induction of host cell death has significant implications on the development of disease and the subsequent immune response. Although *L. monocytogenes* can activate multiple pathways of host cell death, including necrosis, apoptosis, and pyroptosis, like most intracellular pathogens *L. monocytogenes* has evolved a series of adaptations that minimize host cell death to promote its virulence. Understanding how *L. monocytogenes* modulates cell death during infection could lead to novel therapeutic approaches. In addition, as *L. monocytogenes* is currently being developed as a tumor immunotherapy platform, understanding how cell death pathways influence the priming and quality of cell-mediated immunity is critical. This review will focus on the mechanisms by which *L. monocytogenes* modulates cell death, as well as the implications of cell death on acute infection and the generation of adaptive immunity.

## 1. Introduction

*Listeria monocytogenes* is a Gram-positive facultative intracellular pathogen. Due in large part to its ability to survive in both cold and high-salt conditions, it enters the food chain and can lead to the severe disseminated infection Listeriosis [1]. Following ingestion, *L. monocytogenes* can invade intestinal epithelial cells, gaining access to the lymphatic system and blood stream, ultimately resulting in dissemination to the liver, spleen, central nervous system, and, in pregnant women, the placenta. Infection causes symptoms ranging from mild gastroenteritis to more severe meningitis and spontaneous miscarriage in the context of disseminated infections [2]. Disseminated listeriosis can result in mortality rates as high as 30% despite antibiotic treatment [2].

Following ingestion and upon entry into a host cell, either through phagocytosis or internalin-dependent receptor mediated endocytosis [3,4], *L. monocytogenes* utilizes the cholesterol-dependent cytolysin (CDC) listeriolysin O (LLO) to escape the phagosome into the cytosol [5,6]. Once in the cytosol, *L. monocytogenes* expresses the protein ActA to hijack host actin, thus facilitating cell-to-cell spread [7]. The combination of LLO and ActA results in an almost exclusively intracellular lifecycle during infection, thereby avoiding extracellular host defenses, including complement and neutrophils [8,9,10]. Indeed, loss of either LLO or ActA leads to full attenuation of virulence demonstrating the importance of accessing and maintaining its intracellular niche [5,7]. Furthermore, as discussed throughout this review, *L. monocytogenes* has developed multiple strategies to maintain host cell viability and avoid triggering both programmed and non-programmed host cell death pathways to promote its virulence.

In addition to being an important human and animal pathogen, *L. monocytogenes* is also being developed as a novel vaccine platform, particularly in the context of tumor immunotherapy [11]. Due in large part to its constitutive intracellular lifecycle, *L. monocytogenes* infection naturally triggers robust CD8+ T-cell responses [12]. While the exact mechanisms by which *L. monocytogenes* triggers cell-mediated immunity remain unclear, its promise as an immunotherapy platform is illustrated by the >15 active or completed clinical trials using attenuated *L. monocytogenes* for the treatment of a variety of cancers (http://clinicaltrials.gov). *L. monocytogenes* naturally targets antigen-presenting cells during infection and, due to it cytosolic localization, delivers antigens directly to the class I major histocompatibility complex (MHC) presentation pathway. *L. monocytogenes* is also highly genetically tractable, facilitating both pathogen attenuation for clinical safety and the ability to engineer the pathogen to express tumor antigens of interest [11]. Two different *L. monocytogenes*-based immunotherapeutic platforms from Advaxis and Aduro Biotech take advantage of the long standing observation that while cytosolic access is necessary for triggering cell mediated immunity, cell-to-cell spread of the pathogen is not, thereby ensuring vaccine safety [13,14]. Understanding how cell death influences immunity in the context of *L. monocytogenes* infection is important to optimize these platforms for the generation of robust cell-mediated immune responses.

*L. monocytogenes* infection impacts a variety of different host cell death pathways, including both programmed and non-programmed cell death. In this review, we will discuss the influences of host cell death pathways, including necrosis and necroptosis, apoptosis, and inflammasome-mediated pyroptosis on both *L. monocytogenes* virulence as well as *L. monocytogenes*-induced immunity. We will highlight the ways in which these responses are triggered and the mechanisms used by *L. monocytogenes* to manipulate activation of cell death. Understanding how cell death influences both acute infection and *L. monocytogenes* triggered cell-mediated immunity could provide critical insights into novel therapeutics for the treatment of infection, as well as the development of vaccine strains as cancer immunotherapies.

## 2. Necrosis and Necroptosis

Traditionally, necrotic cell death was thought to be an accidental, uncontrolled, lytic, and inflammatory cell death. However, more recently it has become clear that necrosis can also be programmed, most notably in the cell-death pathway called necroptosis, potentially as an antimicrobial defense against intracellular pathogens [15]. Traditional necrosis is triggered by osmotic imbalances and/or the activity of pore forming toxins, whereas the necroptosis pathway is a tightly regulated programmed cell death pathway activated through multiple different signaling cascades ultimately leading to the activation of Receptor Interacting Serine/Threonine-Protein Kinase 3 (RIPK3) kinase and the pseudokinase mixed lineage kinase domain-like, MLKL, the executioner of necroptosis [15,16]. Importantly, necroptosis and apoptosis signaling cascades intimately interact such that inhibition of apoptosis potentiates the necroptosis pathway, potentially as a host defense strategy for pathogens that manipulate apoptotic pathways to promote their virulence [17]. Necrotic death, traditional or programmed, is characterized by organelle damage, pore formation, cellular swelling, and osmotic lysis, ultimately releasing cellular content to the extracellular space, including danger-associated molecular patterns (DAMPs), such as HMGB1 [18,19,20]. Due to the downstream effects of DAMP release, necrosis was originally hypothesized to be an inflammatory and immune-stimulating form of cell death.

The essential *L. monocytogenes* virulence factor listeriolysin O (LLO) is a member of the cholesterol dependent cytolysin family that includes many other important pore forming toxins including pneumolysin and streptolysin O, among others [21]. As such, it is not surprising that LLO has the capacity to induce traditional necrosis; however, the relevance of this to infection is less clear [22,23,24]. *L. monocytogenes* has evolved multiple regulatory mechanisms, including transcriptional, translational and posttranslational regulation, to limit the toxicity of LLO in vivo, thereby ensuring survival of host cells and maintenance of intracellular niches [25,26,27]. Perhaps most notably, LLO contains a series of residues known as the acidic triad that ensures stability at low pH but results in instability at neutral pH. This adaptation ensures limited activity of LLO in the neutral pH of the cytosol, thereby limiting toxicity, but allowing activity in the acidifying environment of the maturing phagosome [28]. Importantly, bacteria with mutations that result in increased production and/or activity of LLO trigger traditional necrosis and are severely attenuated in vivo [25,26,29]. In this context, attenuation is mediated by neutrophils, as the virulence of *L. monocytogenes* expressing toxic LLO is rescued following depletion of neutrophils [25]. Nevertheless, despite the multiple redundant mechanisms for controlling the expression and activity of LLO, and their demonstrated role in promoting virulence, other studies have demonstrated that LLO can still be active at a neutral pH depending on the relative concentration of cholesterol in the target membrane [30]. As such, the potential importance of LLO-mediated necrosis under physiologic LLO expression conditions in vivo is not yet fully understood, in part due to the limited tools available to study non-programmed cell death in vivo.

The role of programmed necrosis during *L. monocytogenes* infection is significantly less clear, as necroptosis is a relatively new field and studies are just beginning to uncover potential roles for programmed necrosis in response to bacterial infections. Recent work from the Orihuela Lab demonstrated that *L. monocytogenes* and other pore-forming, toxin-producing bacteria could induce a RIPK3/MLKL dependent necroptosis in macrophages, although in the case of *L. monocytogenes* whether this was due to extracellular LLO, phagosomal escape, or even the presence of LLO was not assessed [23]. Additionally, the Lecuit Lab recently reported that *L. monocytogenes* infection triggers massive RIPK1-mediated necroptotic depletion of Kupffer Cells in the livers of infected mice [31]. In this model, the induction of Kupffer Cell necroptosis recruits monocytes that more robustly control *L. monocytogenes* infection and then go on to reseed the liver as tissue resident macrophages, demonstrating that in this context the necroptosis pathway is a host protective response [31]. That said, the true impact of *L. monocytogenes*-associated necroptosis on in vivo infection is yet to be determined, as challenge of MLKL-deficient mice with *L. monocytogenes* has not yet been reported. Interestingly, an alternative RIPK3-independent, IRF3-dependent pathway of *L. monocytogenes*-induced, programmed necrosis has recently been reported [32]. The mechanism by which IRF3-dependent necrosis is induced during infection is unknown, though the transcriptional activity of IRF3 itself is not necessary to elicit death. The authors of this study interpreted the increased resistance of IRF3^−/−^ mice to indicate that in this context, programmed necrosis was beneficial to *L. monocytogenes*; however, it is more likely that the resistance of IRF3^−/−^ mice is associated with its role in type I interferon activation, as has also been observed in IFNAR^−/−^ mice [33,34]. Additionally, IRF3^−/−^ mice have been shown to also be deficient in a Bcl-2 family protein [35]. As Bcl-2 family members have known roles in cell death processes, it is likely that deficiency of this molecule contributes to the phenotype.

Both traditional and programmed necrosis are highly inflammatory processes that, in contrast to apoptosis, are thought to be pro-immunogenic. Injection of necrotic cells leads to recruitment of innate immune cells and upregulation of costimulatory molecule expression on antigen-presenting cells [36,37,38], ultimately leading to heightened T cell stimulation and proliferation [38]. In contrast, in the context of *L. monocytogenes* immunization, necrosis inhibits optimal T-cell priming and, ultimately, protective immunity. Immunization with a strain of *L. monocytogenes* engineered to express mis-regulated LLO led to decreased primary and recall CD8+ T-cell responses [39]. Consistent with necrosis being an inflammatory process, however, *L. monocytogenes*-induced necrosis was able to boost immunity induced by primary dendritic cell immunization [39]. The mechanism by which necrosis impairs *L. monocytogenes* stimulated cell-mediated immunity is incompletely understood, though Theisen et al. suggested that hyperactivation of necrosis led to both a loss of cross-presenting dendritic cells as well as suboptimal expression of costimulatory molecules [39]. Cross-priming from CD11c^+^CD8α^+^ expressing dendritic cells is critical for T cell cytotoxic ability and proliferation during *L. monocytogenes* infection [40,41]. Loss of these cell subsets could lead to the diminished protective immune response seen during infection with necrosis inducing strains. Additionally, *L. monocytogenes*-induced necrosis resulted in a loss of CD86 expression on dendritic cells 48 h post infection relative to wild type immunizations [39]. Though still showing larger CD86 expression than PBS immunized mice, deficiencies in co-stimulatory molecule expression compared to wild type immunization could contribute to impaired protective immunity during infection with necrosis-inducing *L. monocytogenes*. Nevertheless, contrary to the proimmunogenic role of necrosis in the context of sterile immunity, activation of necrosis in the context of *L. monocytogenes* infection results in impaired host cell-mediated immunity.

**Figure 1:**
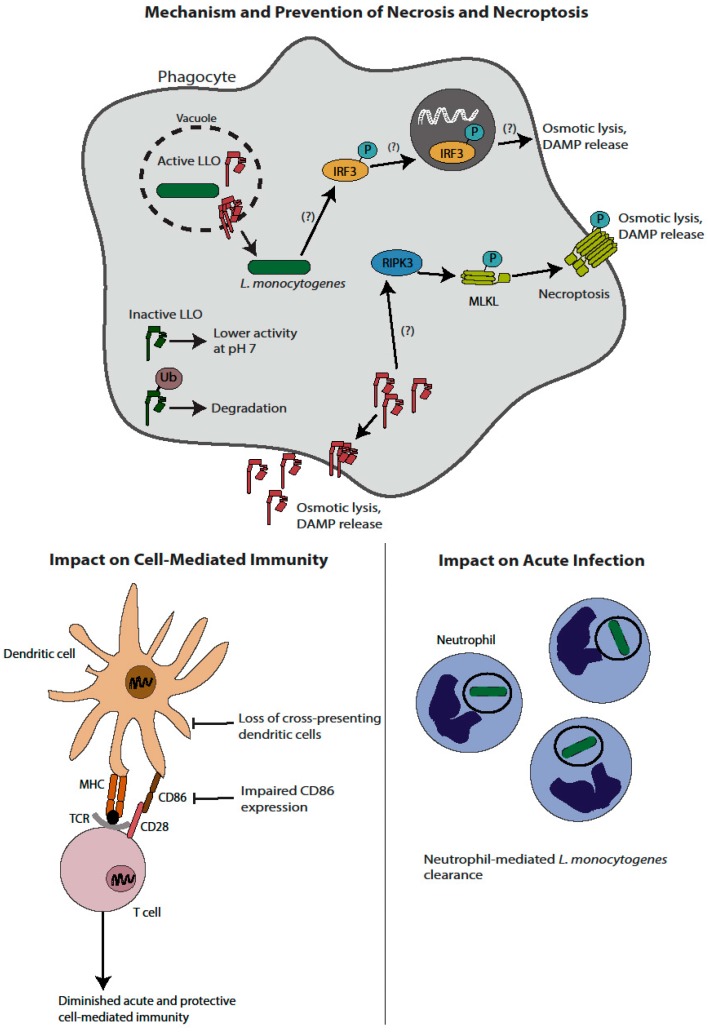
Induction of necrosis by *L. monocytogenes* and implications on immunity and virulence. LLO can induce traditional necrosis; as such, *L. monocytogenes* has evolved mechanisms to avoid lytic activity of LLO outside the vacuole, including an acidic pH optimum and ubiquitin-mediated degradation. Strains of *L. monocytogenes*-induced to express active LLO in the cytosol lead to membrane pore formation and osmotic lysis. In addition, *L. monocytogenes* induces programmed necrosis. Multiple proposed pathways exist, including a RIPK3-mediated pathway leading to pore formation by MLKL and an IRF3 dependent pathway that occurs by a yet undefined mechanism. Induction of necrosis ultimately leads to a host-protective neutrophil-mediated clearance of *L. monocytogenes* and an impaired cell-mediated immune response. Impaired immunity is at least in part mediated by diminished numbers of cross-presenting dendritic cells, as well as lower CD86 expression.

Taken together (Figure 1), activation of traditional necrosis due to LLO-mediated toxicity is associated with both decreased virulence and decreased activation of adaptive immunity. Consistent with these observations, *L. monocytogenes* has evolved multiple mechanisms to regulate LLO expression, translation, and activity to minimize host toxicity. The role of programmed necrosis is less clear, although the preponderance of data suggests that this is a host protective response that *L. monocytogenes* must avoid to promote its pathogenesis, while the role of programmed necrosis in *L. monocytogenes*-triggered immunity is unknown.

## 3. Pyroptosis

Pyroptosis, an alternative form of lytic cell death, is mediated by cytosolic innate immune sensing complexes called inflammasomes [42,43]. Canonical inflammasomes are multiprotein complexes made up of receptors (nucleotide binding domain and leucine-rich repeat containing receptors (NLRs) or absent in melanoma (AIM2)-like receptors (ALRs)), the adaptor protein apoptosis-associated spec-like protein containing a caspase recruitment domain (ASC), and the pyroptosis executioner caspase-1 [44,45,46,47,48]. Multiple types of inflammasomes respond to a variety of pathogen-associated or danger-associated molecular patterns (PAMPs and DAMPs). For example, the NLRC4 inflammasome, which requires neuronal apoptosis inhibitory proteins (NAIPs) as adaptor molecules, recognizes flagellin and type III secretion system components [49], whereas the AIM2 inflammasome recognizes double stranded DNA in the cytosol [50,51]. Despite intense study, the molecular mechanism by which NLRP3 is activated by a diverse array of molecules, including uric acid, ATP, and pore forming toxins, is less well understood [52]. Independent of specific PAMPs/DAMPs and the NLRs/ALRs they activate, downstream signaling is conserved such that ASC is recruited to the receptor, resulting in the recruitment, autoproteolysis, and activation of caspase-1. Caspase-1 activation leads to several downstream effects including maturation and secretion of inflammatory cytokines IL-1β and IL-18, modulation of lipid mediators called eicosanoids, and induction of gasdermin D (GSDMD)-dependent pyroptosis [53,54,55,56]. Pyroptosis shares characteristics of both apoptosis and necrosis; like apoptosis, pyroptosis is characterized by DNA cleavage, nuclear condensation, and caspase dependence [18], whereas similar to necrosis, pyroptosis results in membrane pore formation, subsequent water influx, cellular swelling, and eventual release of cytoplasmic content [57,58]. Additionally, similar to programmed necrosis, there appears to be crosstalk between pyroptosis and apoptosis such that suppression of caspase-1 or GSDMD results in activation of an alternative caspase-8 dependent pyroptosis [59].

Inflammasome activation is a potently host-protective response in the context of a variety of bacterial infections [60], including Listeriosis [39,61,62,63]. The mechanisms by which inflammasome activation protects the host however are not clear, particularly in the context of *L. monocytogenes* infection. As is the case with necrosis, it has been proposed that loss of the intracellular niche and exposure to extracellular neutrophils may result in killing of intracellular pathogens, potentially through the creation of pore-induced intracellular traps (PITs) [61]. However, unlike in the context of necrosis, attenuated inflammasome-activating *L. monocytogenes* are not rescued in the absence of neutrophils [63], suggesting that host protection by inflammasome activation is multifactorial. For example, activation of caspase-1 may be directly antimicrobial by activating GSDMD, which can then bind to cardiolipin in bacterial membranes and cause subsequent pore formation [64]. Consistent with this, supernatants from pyroptotic cells directly reduced colony forming units (CFU) of both Gram-negative and Gram-positive pathogens, including *L. monocytogenes* [64]. Regardless of the mechanism, activation of the inflammasome can potently attenuate virulence, potentially through multiple redundant mechanisms; as such, *L. monocytogenes* has developed strategies to avoid activation of the inflammasome.

*L. monocytogenes* represses flagellin expression in vivo, at least in part via the transcriptional regulator MogR, limiting activation of the NLRC4 inflammasome [65,66]. Misregulation of flagellin expression or forced ectopic expression of flagellin leads to potent virulence attenuation in an NLRC4-dependent manner, highlighting the importance of avoiding inflammasome activation to promote virulence [63,67,68]. Additionally, as bacteriolysis in the cytosol triggers activation of the AIM2 inflammasome [50,51,69,70], *L. monocytogenes* has evolved a variety of mechanisms to ensure cytosolic survival, thus avoiding inflammasome activation and promoting virulence [69,71,72]. Specifically, the protein of unknown function YvcK and its regulatory kinase, PrkA, are required for resistance to cell wall stress, cytosolic survival, AIM2 avoidance, and, ultimately, virulence in vivo [69,71]. Similarly, peptidoglycan modification enzymes that are necessary for lysozyme resistance promote cytosolic survival and AIM2 avoidance [73]. How the host cell targets cytosolic bacteria for killing is largely unknown; however, it is clear that adaptations to protect against these host defenses are essential for AIM2 inflammasome evasion and ultimately virulence. Finally, although it has been demonstrated that pore-forming toxins including LLO can trigger NLRP3 activation [74,75,76], as mentioned above, *L. monocytogenes* regulates LLO transcriptionally, translationally, and post-translationally, to minimize activity on the plasma membrane and therefore minimize activation of the NLRP3 inflammasome in vivo [26,27,28,29].

Similar to necrosis, it was hypothesized that proinflammatory pyroptosis would promote immunity. As *L. monocytogenes* largely avoids inflammasome activation during infection, multiple groups engineered *L. monocytogenes* to hyperactivate the inflammasome in the hopes of promoting increased cell-mediated immune responses [63,68]. Somewhat surprisingly, however, hyper-inflammasome activation significantly reduced cell-mediated immunity [39,63,77]. Importantly, inhibition of immunity due to inflammasome activation is not simply an artifact of hyperactivation, as immunization of caspase-1/11 deficient mice with wild-type *L. monocytogenes* resulted in improved immunity (unpublished observations Erin Theisen, Courtney McDougal, and John-Demian Sauer). This suggests that even the small amount of inflammasome activation during wild type infection inhibits cell-mediated immunity. How inflammasome activation negatively affects *L. monocytogenes*-stimulated immunity is less clear. While the maturation and secretion of proinflammatory cytokines IL-1β and IL-18 does not contribute to the inflammasome-impaired immune response (unpublished observations Erin Theisen, Courtney McDougal, and John-Demian Sauer), it is clear that some other component of the inflammatory milieu inhibits priming of an optimal CD8+ T-cell response [39]. One hypothesis is that the earlier, more robust inflammatory cytokine response associated with inflammasome activation is detrimental to optimal T-cell priming [39,77], consistent with the model that both too much and too little inflammation is detrimental for priming an optimal T-cell response. Alternatively, inflammasome-mediated eicosanoid modulation may negatively impact the generation of cell-mediated immunity in the context of *L. monocytogenes* infection. von Moltke et al. highlighted a COX-dependent hypothermia and vascular leakage in mice after delivery of a potent inflammasome agonist [54]. Additionally, eicosanoids have been implicated in regulating CD8+ T-cell responses in the context of *Mycobacterial* and LCMV-associated immunity [78,79]. Specifically, PGE2 has been reported to inhibit CD8+ T-cell proliferation in some contexts, offering another potential mechanism by which inflammasome activation could inhibit cell mediated immunity [79,80]. Whether or not inflammasome-dependent eicosanoid modulation impacts *L. monocytogenes*-stimulated immunity remains to be determined. Finally, the role of cellular lysis during pyroptosis on immunity is also incompletely understood. Pore formation and lysis can result in release of DAMPs such as HMGB1 modulating the inflammatory milieu associated with inflammasome activation. Previously, separating the impact of pyroptosis from other inflammasome consequences was difficult; however, the discovery of GSDMD as the mediator of pore formation during pyroptosis [55,56] should allow for the assessment of the specific role of pyroptosis in cell-mediated immunity.

**Figure 2:**
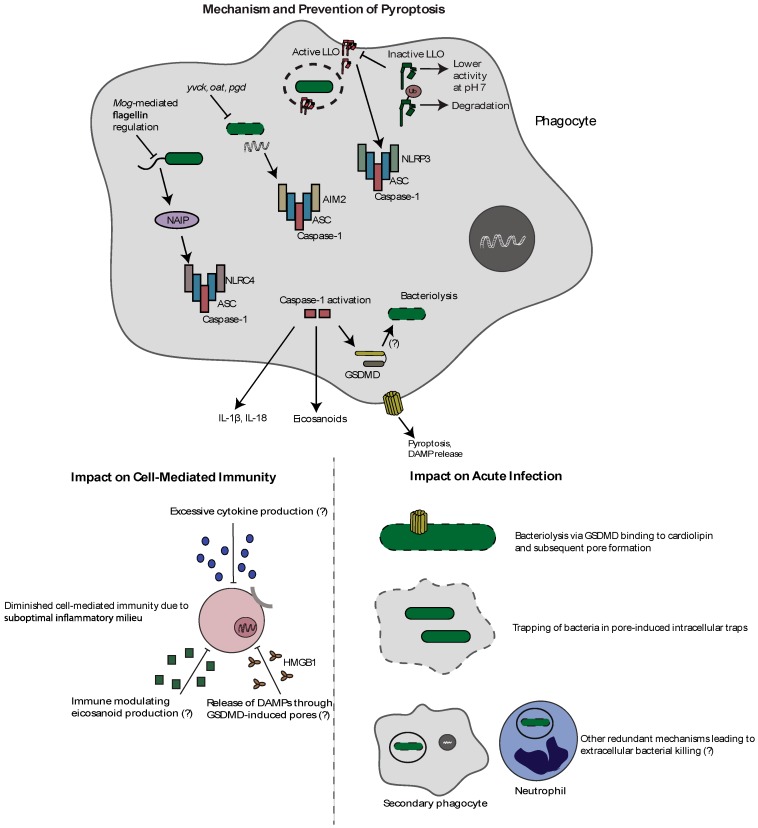
Mechanism and effects of pyroptosis during *L. monocytogenes* infection. *L. monocytogenes* can activate the NLRC4, AIM2, and NLRP3 inflammasomes through flagellin, bacterial DNA release, and LLO-mediated pore formation, respectively. Inflammasome activation results in caspase-1 autoproteolysis followed by cytokine and eicosanoid release, and the induction of the lytic form of cell death called pyroptosis. Importantly, as this leads to the loss of the intracellular niche, *L. monocytogenes* has evolved strategies to avoid inflammasome activation. It represses flagellin during infection through MogR, regulates expression and activity of LLO, and prevents release of bacterial DNA through genes designed to combat cell wall stress (yvcK, oat, pgd). Activation of inflammasomes results in decreased bacterial virulence, potentially through direct bacterial pore formation by GSDMD, bacterial trapping in pore-induced intracellular traps, and potential redundant mechanisms of extracellular killing. Additionally, inflammasome activation impairs cell-mediated immunity through a suboptimal inflammatory milieu associated with proinflammatory cytokines, eicosanoids, and pyroptosis-released DAMPS.

Taken together (Figure 2), similar to necrosis, albeit by different mechanisms, it is clear that inflammasome-mediated pyroptosis negatively impacts both virulence and priming of cell-mediated immunity in the context of *L. monocytogenes* infection. As such, *L. monocytogenes*, like other professional intracellular pathogens [81], has evolved mechanisms to avoid detection by this potent, host-protective, innate immune defense.

## 4. Apoptosis

Apoptosis is a non-lytic cell death characterized by cell shrinkage, chromatin condensation, and extensive membrane blebbing that produces apoptotic bodies [82]. Apoptosis is traditionally subdivided into two pathways: (1) the extrinsic pathway, triggered by ligands such as tumor necrosis factor family molecules which trigger death receptor oligomerization and activation of caspase-8 [83]; or (2) the intrinsic pathway, initiated by signals such as oxidative stress or DNA damage, which ultimately lead to mitochondrial permeabilization by pro-apoptotic Bcl-2 family proteins, release of cytochrome *c* and, ultimately, formation of the apoptosome resulting in the activation of caspase-9 [84]. Both pathways require the activation of downstream caspases, the most important of which is the caspase-3, which leads to a substantial reorganization of the cytoskeleton as well as degradation of chromosomal DNA, inactivation of inflammatory DAMPs, and, ultimately, culminates in the ordered disassembly of the cell into apoptotic bodies [85]. As a natural process during development, apoptosis is not only non-inflammatory but in many cases is actively anti-inflammatory, which has led to the idea that some pathogens have hijacked this system to enhance and perpetuate infection [36,86].

Over 20 years ago it was first reported by Unanue and colleagues that infected hepatocytes undergo apoptosis during *L. monocytogenes* infection. This study suggested that hepatocyte apoptosis was host-protective by recruiting neutrophils to the sites of infection, in contrast to the canonical view of apoptosis as being anti-inflammatory [87]. Since these early studies it has been demonstrated by other groups that infected hepatocytes undergo apoptosis, likely in a TNFα dependent manner [88,89], although whether this is host protective or beneficial to the bacterium remains unclear. Both TRAIL-deficient mice, as well as mice deficient in a variety of proapoptotic BH3-only Bcl-2 family members, were significantly more resistant to *L. monocytogenes* challenge, although this was not definitively localized to the hepatocyte compartment [90,91]. While it is clear that hepatocytes can undergo apoptosis during infection, the mechanism by which this is mediated and the consequences of hepatocyte apoptosis on the outcome of *L. monocytogenes* infection remains to be fully defined.

While hepatocyte apoptosis in the context of *L. monocytogenes* infections has been only sporadically documented, apoptosis of lymphocytes early during infection is a well-established consequence of *L. monocytogenes* infection. It has been long understood that infection leads to a depletion of lymphocytes within the periarteriolar lymphoid sheaths [92], seen as early as 24 h post infection in a dose-dependent manner [93]. In contrast to apoptosis of infected hepatocytes, lymphocyte apoptosis is not associated with direct infection of these cells, but rather, infected phagocytes are in close proximity to the uninfected apoptotic lymphocytes [93,94]. Two, non-mutually exclusive pathways have been implicated in triggering lymphocyte apoptosis during *L. monocytogenes* infection: (1) the direct activity of sub-lytic concentrations of LLO on lymphocytes and (2) the copious amounts of type I interferon produced in the context of *L. monocytogenes* infection. One model for LLO-mediated apoptosis is that it acts as a perforin alternative for the delivery of host granzyme to target lymphocytes [95,96,97]. Ex vivo isolation of CD4^+^ T cells treated with exogenous LLO led to activation of apoptotic caspases-3, -6, and -9, and subsequently 75% T cell apoptosis by 8 h post treatment [95], whereas apoptosis of granzyme-deficient T cells was reduced by approximately 50%. Importantly, this seminal work was done using CD4^+^ T cells that only had ~30% staining positive for granzymes, suggesting an even larger potential role for granzyme in lymphocyte apoptosis [95]. Consistent with this model, granzyme-deficient mice demonstrated less lymphocyte apoptosis and were 15-fold more resistant to acute *L. monocytogenes* infections [95], suggesting that activation of lymphocyte apoptosis is a pathogen beneficial virulence strategy.

In addition to a direct role for extracellular LLO, type I interferons (IFN) have been implicated in lymphocyte apoptosis during *L. monocytogenes* infections. *L. monocytogenes* infection results in robust production of type I interferon due to activation of STING by both c-di-AMP and less frequently cytosolic bacterial DNA [98,99,100]. Type I IFN subsequently has been shown to antigen-independently preactivate T cells, potentially increasing T cell sensitivity to apoptosis [101]. Following systemic infection with *L. monocytogenes*, type I IFN receptor (IFNAR)-deficient mice demonstrate both decreased lymphocyte apoptosis and increased resistance to infection, consistent with a model whereby lymphocyte apoptosis is a pathogen-driven process to promote virulence [33,34,101]. Independent of the mechanism of apoptosis induction, it is thought that the increased virulence of *L. monocytogenes* associated with lymphocyte apoptosis is mediated by the activation of an immunosuppressive state. Uptake of apoptotic bodies results in production of anti-inflammatory cytokine production, notably IL-10, by phagocytes [102]. IL-10 antagonizes production of proinflammatory IFN-γ, which is required for optimal acute defense against *L. monocytogenes*. Consistent with this model, IL-10-deficient mice showed increased resistance to *L. monocytogenes* infection despite having equivalent levels of apoptotic splenic cells, suggesting the susceptibility associated with lymphocyte apoptosis could be mediated by IL-10 [94,103]. Finally, mice lacking lymphocytes altogether are acutely resistant to *L. monocytogenes*, further suggesting that increased lymphocyte apoptosis results in decreased resistance to infection [103,104,105].

In addition to effects on acute virulence, induction of apoptosis during *L. monocytogenes* infection influences the priming of adaptive immune responses. Perhaps not surprisingly, given the known detrimental role of IL-10 in the generation of cell mediated immunity [103], *L. monocytogenes* engineered to induce apoptosis generated worse T-cell responses following immunization compared to wild type *L. monocytogenes* [39]. Somewhat surprisingly, however, in this context, increased apoptosis was not associated with increased levels of IL-10 suggesting additional, apoptosis-dependent, IL-10 independent detrimental effects on cell mediated immunity. How, mechanistically speaking, apoptosis of either infected cells or bystander lymphocytes might negatively influence the generation of cell-mediated immunity independent of IL-10 is currently unknown. Also consistent with a negative impact of increased apoptosis on generation of adaptive immunity was the observation that hyperinduction of type I interferons is detrimental to CD8+ T-cell priming, although this was never explicitly tied to levels of lymphocyte apoptosis [106].

**Figure 3:**
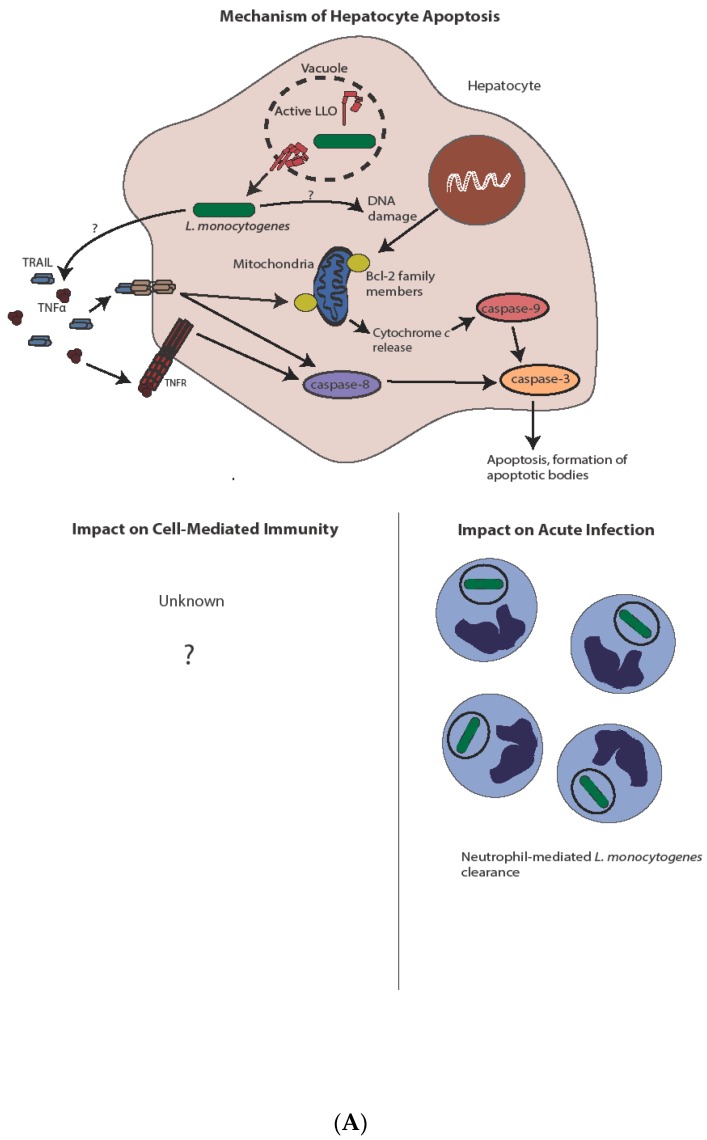

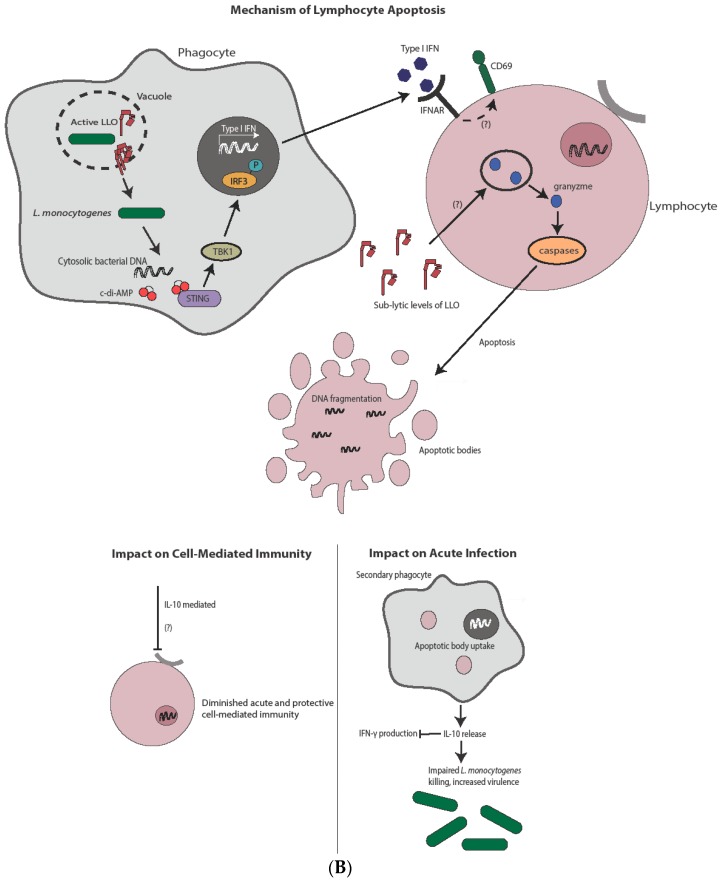
Mechanism and impact of apoptosis during *L. monocytogenes* infection. (**A**) Direct infection of hepatocytes with *L. monocytogenes* leads to apoptosis, potentially through a TNFα and/or Bcl-2 family member-mediated mechanism. Induction of apoptosis through either pathway ultimately leads to caspase-3 activation. Hepatocyte apoptosis is host-protective through release of chemoattractants and subsequent neutrophil-mediated *L. monocytogenes* killing. The complete impact on cell-mediated immunity, if any, is unknown. (**B**) In contrast to hepatocytes, *L. monocytogenes*-mediated lymphocyte killing is independent of direct infection. Lymphocyte apoptosis is instead induced through sublytic concentrations of LLO and/or type I IFN from infected phagocytes. Type I IFN produced after STING activation leads to antigen-independent preactivation of T cells as indicated by CD69 expression, potentially sensitizing them to apoptotic capabilities of LLO. Uptake of apoptotic bodies by secondary phagocytes leads to increased bacterial virulence though IL-10 production, as well as diminished cell-mediated immunity, through both IL-10-dependent and potentially IL-10-independent mechanisms.

In summary (Figure 3), *L. monocytogenes*-induced lymphocyte apoptosis, whether mediated by LLO, type I interferons, or a combination of the two, potentiates acute infection, likely through the induction of an anti-inflammatory state induced by uptake of apoptotic bodies. Indeed, Ucker and colleagues demonstrated that direct injection of apoptotic cells, but not necrotic cells, increases virulence of *L. monocytogenes* by a mechanism they coined “innate apoptotic immunity” [107]. It is exciting to speculate that this is an adaptation evolved by *L. monocytogenes* to promote its virulence. Promoting apoptosis to potentiate virulence is a strategy used by some other pathogens, including HIV, whereby CD4^+^ T cell apoptosis inhibits clearance of the virus [108], while *Mycobacterium avium*-induced apoptosis mediates bacterial spread [109]. The mechanism of induction of hepatocyte apoptosis is significantly less well understood, as is its impact on acute infection or immunity. Finally, the detrimental effect of apoptosis on *L. monocytogenes*-stimulated immunity suggests that mechanisms that might inhibit apoptosis could result in more robust *L. monocytogenes*-based immunotherapy platforms.

## 5. Conclusions and Future Directions

In summary, cell death has potent impacts on the outcomes of infections, particularly in the context of exquisitely adapted intracellular pathogens such as *L. monocytogenes*. In most cases, host cell death is protective and *L. monocytogenes* has evolved ways to avoid activation of these pathways through regulation of their virulence factors. Whether *L. monocytogenes* has specific virulence factors to directly modulate cell death pathways is an outstanding question, but as more cell death pathways, such as necroptosis, are discovered, that possibility becomes more and more likely. The exception to the cell death avoidance model is the activation of bystander apoptosis of lymphocytes. Importantly, this form of cell death does not directly eliminate a replication niche and in fact appears to be detrimental to the host and beneficial to the pathogen, fueling the longstanding, speculative, but exciting hypothesis that *L. monocytogenes* promotes type I interferon responses to create an immunosuppressive environment to enhance virulence. Finally, our understanding of how cell death influences the generation of adaptive immune responses is still in its infancy. The classic dogma of immunogenic necrosis and tolerogenic apoptosis has been turned on its head, even in the context of sterile immunity. How these pathways influence immunity in the context of infection is a future frontier. Furthermore, as more novel cell death pathways continue to be discovered, their interactions with the innate and adaptive immune systems will have to be understood in order to refine our use of bacteria such as *L. monocytogenes* as beneficial microbes in the context of vaccines and tumor immunotherapy.
